# Analysis of the global transcriptome and miRNAome associated with seed dormancy during seed maturation in rice (*Oryza sativa* L. cv. Nipponbare)

**DOI:** 10.1186/s12870-024-04928-6

**Published:** 2024-03-26

**Authors:** Minsu Park, Sang-Yoon Shin, Hongman Moon, Woochang Choi, Chanseok Shin

**Affiliations:** 1 Department of Agricultural Biotechnology, Seoul National University, Seoul, 08826 Republic of Korea; 2 Research Institute of Agriculture and Life Sciences, Seoul National University, Seoul, 08826 Republic of Korea; 3 Plant Genomics and Breeding Institute, Seoul National University, Seoul, 08826 Republic of Korea; 4 Research Center for Plant Plasticity, Seoul National University, Seoul, 08826 Republic of Korea

**Keywords:** Rice, Seed dormancy, Seed maturation, Plant hormone, Transcriptome, miRNAome

## Abstract

**Background:**

Seed dormancy is a biological mechanism that prevents germination until favorable conditions for the subsequent generation of plants are encountered. Therefore, this mechanism must be effectively established during seed maturation. Studies investigating the transcriptome and miRNAome of rice embryos and endosperms at various maturation stages to evaluate seed dormancy are limited. This study aimed to compare the transcriptome and miRNAome of rice seeds during seed maturation.

**Results:**

*Oryza sativa* L. cv. Nipponbare seeds were sampled for embryos and endosperms at three maturation stages: 30, 45, and 60 days after heading (DAH). The pre-harvest sprouting (PHS) assay was conducted to assess the level of dormancy in the seeds at each maturation stage. At 60 DAH, the PHS rate was significantly increased compared to those at 30 and 45 DAH, indicating that the dormancy is broken during the later maturation stage (45 DAH to 60 DAH). However, the largest number of differentially expressed genes (DEGs) and differentially expressed miRNAs (DEmiRs) were identified between 30 and 60 DAH in the embryo and endosperm, implying that the gradual changes in genes and miRNAs from 30 to 60 DAH may play a significant role in breaking seed dormancy. Gene Ontology (GO) and Kyoto Encyclopedia of Genes and Genomes (KEGG) pathway analyses confirmed that DEGs related to plant hormones were most abundant in the embryo during 45 DAH to 60 DAH and 30 DAH to 60 DAH transitions. Alternatively, most of the DEGs in the endosperm were related to energy and abiotic stress. MapMan analysis and quantitative real-time polymerase chain reaction identified four newly profiled auxin-related genes (*OsSAUR6/12/23/25*) and one ethylene-related gene (*OsERF087*), which may be involved in seed dormancy during maturation. Additionally, miRNA target prediction (psRNATarget) and degradome dataset (TarDB) indicated a potential association between osa-miR531b and ethylene biosynthesis gene (*OsACO4*), along with osa-miR390-5p and the abscisic acid (ABA) exporter-related gene (*OsMATE19*) as factors involved in seed dormancy.

**Conclusions:**

Analysis of the transcriptome and miRNAome of rice embryos and endosperms during seed maturation provided new insights into seed dormancy, particularly its relationship with plant hormones such as ABA, auxin, and ethylene.

**Supplementary Information:**

The online version contains supplementary material available at 10.1186/s12870-024-04928-6.

## Background

Seed development comprises two significant phases: zygotic embryogenesis and seed maturation. Seed maturation is initiated upon the completion of embryogenesis [1]. During maturation, seeds acquire various physiological characteristics, such as dormancy, a crucial adaptive trait in plants, which refers to the innate ability of mature seeds to temporarily suspend germination even under favorable environmental conditions [1, 2]. This adaptive trait allows plants to synchronize their germination with optimal growth conditions, ensuring the survival and successful establishment of the next generation [2, 3]. For several major crops and horticultural plants, seed dormancy is also considered an important agronomic trait that largely affects the overall productivity and the product quality. Failure in the control of seed dormancy against various environmental conditions before harvest leads to pre-harvest sprouting (PHS), which is detrimental to the overall agricultural business and food security in the face of the recent drastic global climate changes [4]. Seed dormancy is controlled by intricate molecular mechanisms involving the interplay of various factors, including plant hormones (phytohormones) [2, 5]. Among the plant hormones, abscisic acid (ABA) and gibberellic acid (GA) have been extensively studied and recognized as key regulators that antagonistically regulate seed dormancy and germination [6, 7]. Additionally, auxin and ethylene are reported as important players in seed dormancy and germination [6].

Auxin, specifically indole-3-acetic acid (IAA), is a pivotal phytohormone involved in numerous aspects of plant growth and development [8]. A previous study revealed that the interaction between *AUXIN RESPONSE FACTOR 10/16* (*ARF10/16*) and ABA response transcription factor *ABSICISIC ACID INSENSITIVE3* (*ABI3*) is crucial for regulating seed dormancy in *Arabidopsis*. In the presence of elevated auxin levels, *ARF10* and *ARF16* trigger *ABI3* transcription, thereby maintaining seed dormancy [9]. In addition, SMALL AUXIN UP RNAs (SAURs), another type of auxin-responsive genes, regulate plant growth and germination in *Arabidopsis* [10, 11]; the *saur32* mutant showed accelerated germination and lower accumulation of ABA compared to the wild-type (WT) *Arabidopsis* [11]. Taken together, these findings indicate that ABA and auxin are closely related in seed dormancy. In addition, they suggest that the SAUR genes can positively control seed dormancy through the interaction between auxin and ABA.

Ethylene, the gaseous phytohormone, can promote seed germination by counteracting ABA signaling [12, 13]. Ethylene signaling controls numerous ethylene response factors (ERFs), which belong to the plant-specific transcription factor families [14, 15]. Studies related to ERF and germination have shown that knockdown of the *AtERF7* exhibited reduced seed germination in response to ABA compared to WT *Arabidopsis* [16]. Overexpression of the *SlERF2* gene showed premature seed germination and less ABA sensitivity compared to WT in tomatoes (*Solanum lycopersicum*) [17]. These results suggest that ERF genes act as positive regulators of germination related to the interaction between ethylene and ABA.

In addition to various phytohormones, evidence suggests that small RNAs, especially microRNAs (miRNAs), regulate seed germination and dormancy [18–21]. A previous study showed that changes in miR156 and miR172 expression affect seed dormancy level of the seed, and miRNA biogenesis-related genes are regulated by the key seed dormancy regulator, *DOG1* [19]. Furthermore, several reported miRNAs regulate seed germination under specific abiotic stress conditions [22–24]. Several studies profiling miRNAs in the developing embryo using small RNA-Seq reported the accumulation of candidate seed dormancy-regulating miRNAs during seed development [25, 26]. The small RNA-mediated non-canonical RNA-directed DNA methylation pathway can promote seed dormancy via the epigenetic suppression of paternal *ALLANTOINASE* allele, which is enhanced by cold treatment [20]. In our previous study, we also reported several candidate miRNAs that exhibited differential expression among rice cultivars with different seed dormancy levels [27]. These studies suggest that miRNA and other small RNAs play key roles in regulating seed germination and dormancy as a top-tier regulator.

While there have been several studies profiling the transcriptome of rice embryos and endosperms, the primary focus of those studies has been on providing transcriptome-wide insights into the relatively early stages of seed development [28, 29]; hence, there is a dearth of studies investigating the transcriptome and miRNAome of rice embryos and endosperms focusing on seed dormancy at various maturation stages. Therefore, this study aimed to profile the dynamics of genes and miRNAs associated with plant hormones that control seed dormancy during maturation.

## Results

### Difference in Nipponbare seed dormancy level according to the maturation stages

**Fig. 1:**
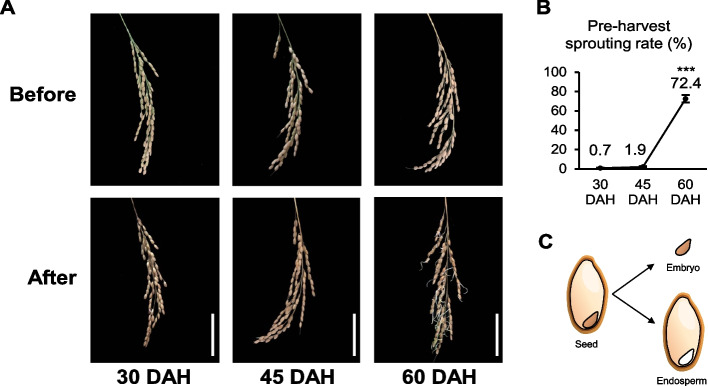
Pre-harvest sprouting (PHS) assay in the Nipponbare seeds and the sampling scheme. **A** The PHS phenotypes of the Nipponbare panicles at 30, 45, and 60 days after heading (DAH). The phenotypes, both before and after PHS assay, are presented. Scale bar, 5 cm. **B** The PHS rates of the Nipponbare panicles at 30, 45, and 60 DAH (*N* = 9). The significance was determined using the Student’s *t-*test, ***P < 0.001. **C** The sampling scheme employed in this study: embryo and endosperm

PHS assays were performed on Nipponbare panicles at 30, 45, and 60 days after heading (DAH) to assess the change of seed dormancy level during maturation. Panicles sampled at 30 and 45 DAH exhibited low PHS rates; however, the rate of that sampled at 60 DAH was approximately 38 times higher than that sampled at 45 DAH (Fig. 1A, B). Results from the PHS assessment indicated that the high seed dormancy level was strongly maintained until 45 DAH, followed by a decrease between 45 and 60 DAH. Therefore, we decided to analyze the transcriptome and small RNAome of the rice seeds to investigate the dynamics of the transcripts and small RNAs associated with the differences in seed dormancy levels across the three maturation stages. Hence, embryos and endosperms from Nipponbare seeds were separately sampled at each maturation stage, and the transcriptome and small RNAome analyses were conducted in the current study (Fig. 1C).

### Marked differences in transcriptional changes in the embryo and endosperm during the various maturation stages

**Fig. 2:**
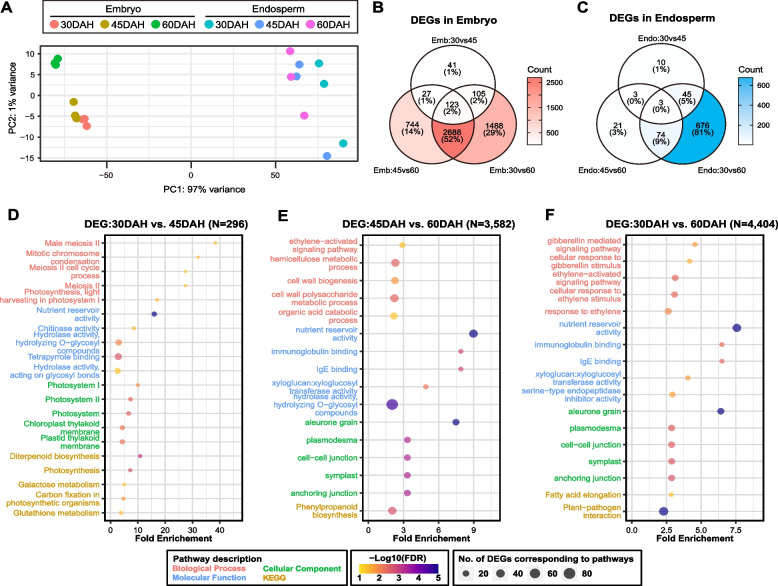
Biological function analysis of differentially expressed genes (DEGs) in the embryo and endosperm. **A** Principal component analysis (PCA) results from transcriptome of the embryo and endosperm at 30, 45, and 60 DAH. Data for each maturation stage of the embryo and endosperm are represented in different colors; biological replicates of samples from the same maturation stage are depicted in the same color. **B**, **C** Venn diagram of the relationship based on the number of DEGs during seed maturation stages in the embryo (**B**) and endosperm (**C**). 30 vs. 45: DEGs between 30 and 45 DAH. 45 vs. 60: DEGs between 45 and 60 DAH. 30 vs. 60: DEGs between 30 and 60 DAH. **D**, **E**, **F** Gene Ontology (GO) and Kyoto Encyclopedia of Genes and Genomes (KEGG) enrichment terms based on DEGs between 30 and 45 DAH (**D**), 45 and 60 DAH (**E**), and 30 and 60 DAH (**F**). The Y-axis represents the enriched GO and KEGG pathway terms. The X-axis represents the amount of fold enrichment of GO and KEGG pathway terms. The top five GO terms associated with “biological process”, “molecular function”, and “cellular component”, and the top five KEGG terms based on fold enrichment > 2 and false discovery rate (FDR) < 0.05 were selected. The enriched KEGG pathway terms were obtained using ShinyGO

The results of the principal component analysis (PCA) from the transcriptomes in the embryo and endosperm revealed variations in the transcript expression patterns between tissues and between maturation stages (Fig. 2A). Particularly, embryo replicates from each maturation stage showed a consistent pattern, with the 60 DAH embryo sample exhibiting a distinct pattern compared with those at 30 and 45 DAH (Fig. 2A). The endosperm replicates did not exhibit a consistent pattern at each maturation stage, suggesting that the stability of embryo transcripts at each maturation stage was higher than that of the endosperm transcripts across the 360 sampled seeds at each maturation stage (in three replicates; 120 seeds per one replicate; Fig. 2A) Results from the following differential gene expression analysis showed that the transcriptome in the embryo exhibited more diverse changes than that in the endosperm across the seed maturation stages (Fig. 2B, C). In total, 5,216 and 832 differentially expressed genes (DEGs; log_2_ fold change (log_2_FC) > 1, p.adj < 0.05) were observed in the embryo and endosperm, respectively, during maturation (Fig. 2B, C). The DEGs between 30 and 60 DAH (“30 vs. 60”) were more abundant than those in the other comparison groups (“30 vs. 45” and “45 vs. 60”) in both the embryo and endosperm (Fig. 2B, C). Thus, the gene expression had a greater tendency to gradually increase or decrease during maturation from 30 to 60 DAH. Notably, the differences observed in the PCA plots and DEGs across the three maturation stages indicated significant alterations in the transcripts and gene expression in the embryo compared with those in the endosperm during seed maturation, particularly from 45 to 60 DAH and 30 to 60 DAH (Fig. 2A-C).

Gene Ontology (GO) and Kyoto Encyclopedia of Genes and Genomes (KEGG) enrichment analyses were performed to gain insights into the biological functions and pathways of DEGs in the embryo and endosperm that may contribute to changes in dormancy levels across the maturation stages. The top five GO terms from “biological process”, “molecular function”, and “cellular component”, and the top five KEGG pathways terms (fold enrichment > 2, false discovery rate (FDR) < 0.05) enriched in the DEGs of the embryo and endosperm are selectively illustrated in Fig. 2D-F and Additional file 1: Fig. S1, respectively. The enriched GO terms for “biological process” and KEGG terms from the DEGs between 30 and 45 DAH in the embryo were associated with meiosis, mitosis, and photosynthesis (Fig. 2D), indicating that the embryo, which is green in color, utilizes chlorophyll to accumulate energy for cell growth (similar to meiosis and mitosis) via photosynthesis during the early maturation stages. The GO terms associated with “biological process” for DEGs between 45 and 60 DAH in the embryo were related to ethylene signaling and cell wall biogenesis (Fig. 2E). In addition, the DEGs between 30 and 60 DAH demonstrated GO terms related to GA and ethylene signaling (Fig. 2F). These results suggest that the DEGs that change in the later maturation stage (45 to 60 DAH) and those that change gradually from 30 to 60 DAH are closely related to hormones involved in seed maturation and dormancy. Based on the “molecular function” and “cellular component” GO and KEGG terms in the embryo, the embryo may serve as a vital component of the seed and play a significant role as an energy storage organ, distinct from seed dormancy (Fig. 2D-F). Unlike the GO and KEGG terms for the embryo, most of the terms for the DEGs in the endosperm were related to energy and abiotic stress (Additional file 1: Fig. S1), indicating that the endosperm is a supplier of nutrients and may not play a direct role in seed maturation and dormancy. All the GO and KEGG terms in the embryo and endosperm are listed in Additional file 2: Tables S1-S6.

### Functional profiling for DEGs that constitute the largest portion of the embryo DEGs and exhibit decreased expression during seed maturation

**Fig. 3:**
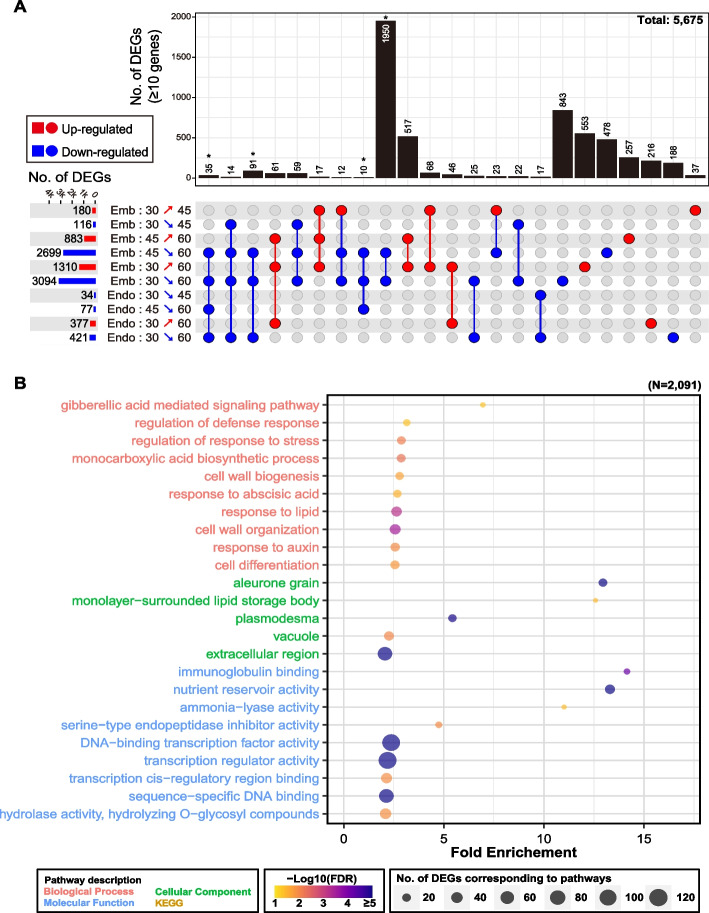
Biological function analysis of the most commonly altered DEGs in UpSet plots. **A** The UpSet plot shows the number of shared DEGs between the tissues and the maturation stages. The horizontal bar graphs and written numbers on the left of intersection matrix represent the numbers of DEGs between two compared conditions. The X-axis in the upper graph represents the number of DEGs corresponding to the lower filled dots, with counts of fewer than 10 DEGs excluded. The sets of connected filled dots indicate a specific intersection of DEGs among the maturation stages in the embryo and endosperm. Red bars, arrows, and dots indicate the up-regulated DEGs, while the blue bars, arrows, and dots indicate the down-regulated DEGs. **B** GO terms based on 2,091 DEGs down-regulated from 45 to 60 DAH and 30 to 60 DAH in the embryo. The Y-axis indicates the enriched GO terms, and the X-axis indicates the amount of fold enrichment of the GO terms. The top ten GO terms associated with “biological process”, “molecular function”, and “cellular component”, based on fold enrichment > 2 and FDR < 0.05 were selected. The KEGG pathway terms were not identified via the KEGG enrichment analysis

Owing to the tendency for a gradual change in the expression levels of the DEGs in the embryo and endosperm during maturation, we categorized the DEGs based on their differential expression patterns across the three maturation stages and visualized them using the UpSet plot (Fig. 3A, Additional file 3: Dataset. S1). In the embryo, a total of 1,950 genes, which represents the highest number of DEGs in the UpSet plot, exhibited consistent down-regulation during two transitions: 45 to 60 DAH and 30 to 60 DAH (Fig. 3A). The DEGs of endosperm were fewer than those of embryo across three maturation stages, and a significant portion (> ~ 30.4%) of them were also up- or down-regulated in the embryo. These results indicate that the transcriptome in the embryo of the Nipponbare seed undergoes major changes during maturation, whereas that in the endosperm exhibits lesser dynamics.

The 2,091 DEGs in the embryo, which included 1,950 down-regulated DEGs described above and another 141 genes that exhibited consistent down-regulation in the embryo and endosperm during 45 to 60 DAH and/or 30 to 60 DAH, were further investigated in the GO and KEGG enrichment analyses (Fig. 3B). Enriched GO terms related to phytohormone responses, including GA, ABA, and auxin, were identified, similar to those depicted in Fig. 2E and F for the embryo DEGs. There were six DEGs, *OsSLRL1* (Os01g0646300), *OsWRKY71* (Os02g0181300), *OsbHLH079* (Os02g0705500) and three F-box domain containing proteins in the GO term “gibberellic acid mediated signaling pathway”. Among them, *OsSLRL1* and *OsWRKY71* are known to act as repressors during GA signaling [30, 31]. The *OsSLRL1* and *OsWRKY71* were found to be reduced during 45 to 60 DAH in the embryo (Additional file 2: Table S7). The expression dynamics of the GA signaling repressors appeared to be correlated with the increase in the PHS rate (Fig. 1B). In the “response to abscisic acid” pathway, among eighteen genes, we found *OsEm1* (Os05g0349800), *OsPP2C30* (Os03g0268600) and *OsCPK21* (Os08g0540400), that were involved in the positive regulation of the ABA signaling pathway [32–34]. Similar to the above-mentioned GA repressors, the down-regulation of these genes may be closely associated with the weakening of the seed dormancy level at 60 DAH in the embryo (Additional file 2: Table S7). Consistent with the down-regulation of these positive regulators of ABA signaling, a rice ABA influx carrier, *OsPM1* (Os05g0381400), was identified [35]; moreover, the predicted rice homolog of the ABA efflux carrier *AtABCG25*, *OsABCG27* (Os11g0177400) [36], was significantly down-regulated during the same period in the embryo (Additional file 3: Dataset S1). These changes imply the overall reduction of ABA-mediated signals in the embryo between 45 and 60 DAH. In the “response to auxin” pathway, seven *SAURs* and four *ARFs* were confirmed. Thus, the transcriptome dynamics during the 45 to 60 DAH transition strongly suggested the complexed cross-talk between various phytohormone-mediated signaling pathways.

### Identification of auxin- and ethylene-related genes potentially responsible for Nipponbare seed dormancy during maturation

**Fig. 4:**
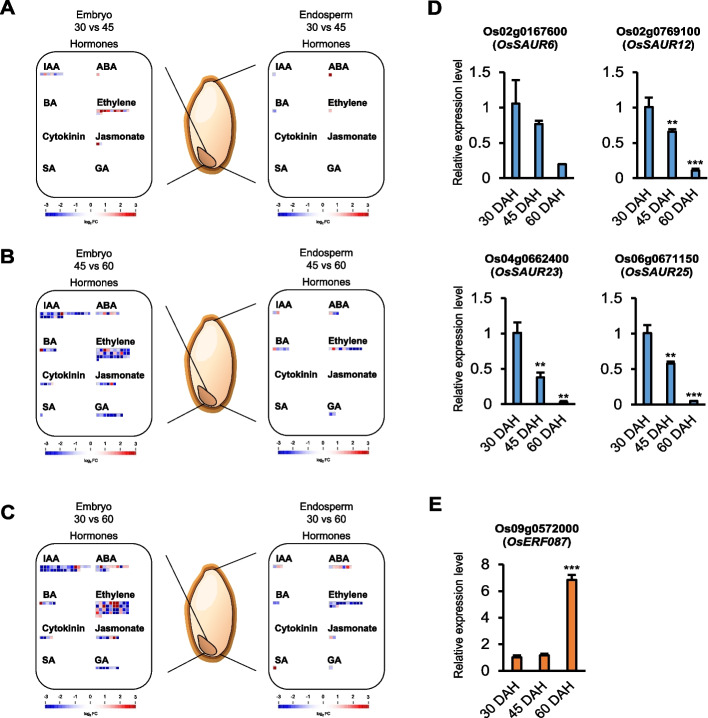
Profiling of genes expected to be involved in seed dormancy during maturation. **A**-**C** Identification of hormone-related DEGs between 30 and 45 DAH (**A**), 45 and 60 DAH (**B**), and 30 and 60 DAH (**C**) in the embryo and endosperm using MapMan analysis. The “Regulation overview” of the MapMan analysis was employed, and the figures were adapted from Additional file 1: Fig S2 for DEGs related to hormones. 30 vs 45: DEGs between 30 and 45 DAH. 45 vs 60: DEGs between 45 and 60 DAH. 30 vs 60: DEGs between 30 and 60 DAH. **D**, **E** The graph displays the relative expression levels of auxin-related genes (**D**) and an ethylene-related gene (**E**) using quantitative real-time polymerase chain reaction. Data are represented as the mean ± standard error of mean (SEM; *N* = 3). The significance was determined using Student’s *t*-test, **P < 0.01 and ***P < 0.001

The DEGs identified in the embryo and endosperm were further analyzed to visualize their functions and expression patterns using MapMan software, which integrates multiple omics data of plants [37]. We focused on hormone-related DEGs identified in the “Regulation overview”. Plant hormones tightly regulate seed dormancy [6, 7], and the DEGs in the embryo mainly represented hormone-related GO terms (Figs. 2E, F and 3B). Analysis of the hormone-related DEGs revealed greater changes, particularly in IAA-, and ethylene-related genes in the embryo compared with those in the endosperm (Fig. 4A-C). The GO terms and MapMan analysis results (Figs. 2E, F, 3B and 4A-C) for embryo and endosperm suggested that the hormone-related DEGs in the embryo played a crucial role in seed dormancy during seed maturation. The hormone-related DEGs of embryo and endosperm are listed in Additional file 2: Tables S8-S13. The “Regulation overview” in all DEGs of the embryo and endosperm are shown in Additional file 1: Fig. S2.

Among the hormone-related DEGs, IAA-, and ethylene-related genes potentially involved in seed dormancy during maturation were specifically selected. The expression levels of the selected *SAUR* and *ERF* genes were validated using quantitative real-time polymerase chain reaction (qRT-PCR; Fig. 4D, E). Based on previous study [11], we focused on *OsSAUR6*, *OsSAUR12*, *OsSAUR23*, and *OsSAUR25*, which exhibited a gradual decrease in expression from 30 to 60 DAH in the embryo (Fig. 4D), indicating their potential role in positively regulating seed dormancy. In contrast, *ERF* genes involved in ethylene signaling are suggested to negatively regulate seed dormancy [16, 17]. The expression level of *OsERF087* was highest at 60 DAH in the embryo, coinciding with the highest PHS rate in the current study (Figs. 1B and 4E). These results suggested that auxin- and ethylene-related genes, including the newly discovered *OsSAUR6*, *OsSAUR12*, *OsSAUR23*, *OsSAUR25*, and *OsERF087*, may significantly affect seed dormancy during maturation.

### Gradual changes in the expression levels of miRNAs during seed maturation

**Fig. 5:**
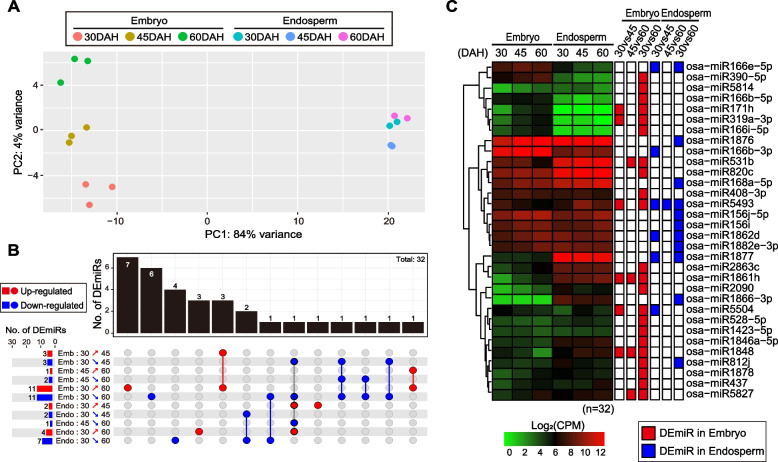
The expression dynamics of microRNAs (miRNAs) after 30 DAH in the embryo and endosperm. **A** The PCA results showed that the miRNA population exhibit more dynamic changes in embryo than in endosperm during seed maturation. PCA plot was drawn after regularized logarithm transformation of the normalized miRNA counts. **B** Visualization of the UpSet plot for the numbers of differentially expressed miRNAs (DEmiRs) and intersections between the testing sets. The horizontal bar graphs and written numbers on the left of intersection matrix represent the numbers of DEmiRs between two compared conditions. The vertical bar graphs and written numbers from above intersection matrix represents the size of the intersections. **C** Heatmap representation of hierarchically clustered DEmiRs during rice seed maturation in the embryo and endosperm. Hierarchical clustering was conducted after log2-transformation of the counts per million (CPM)-normalized miRNA read counts. Boxes colored in red or blue indicate differentially expressed DEmiRs (p.adj < 0.05) between the two sampling timepoints, which are labeled on top of boxes

Small RNA-Seq analysis was conducted from the embryo and endosperm of Nipponbare seeds collected at 30, 45, and 60 DAH to profile the expression dynamics of the miRNA population during rice seed maturation in the embryo and endosperm. First, we confirmed that the expression levels of miRNAs in the embryo exhibited more variances than those in the endosperm during maturation using PCA (Fig. 5A). Consistent with this observation, a differential expression analysis of the embryo samples between 30 and 60 DAH revealed higher numbers of differentially expressed miRNAs (DEmiRs) than those in the endosperm samples (Fig. 5B, C, Additional file 3: Dataset S2).

### Several miRNAs in embryo are predicted to target phytohormone signaling-related genes

**Fig. 6:**
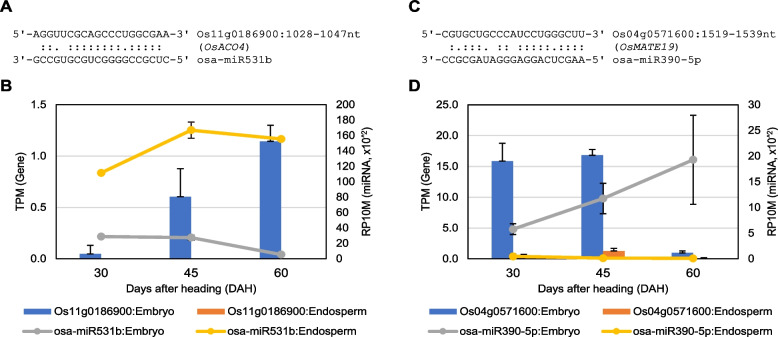
Predicted target genes of DEmiRs in the embryo involved in ethylene and ABA signaling-related genes. **A**, **C** Schematic representation of the pairing between two DEmiRs and their target genes. **B**, **D** Profiled expression levels of the DEmiRs and their predicted target genes in this dataset. TPM: transcript per million mapped, RP10M: reads per ten million mapped

The target genes of DEmiRs were predicted using psRNATarget [38] to investigate the roles of the DEmiRs and their affected biological pathways in the embryo and endosperm during seed maturation. Among the predicted target genes, we further selected DEGs exhibiting anti-correlated changes in their expression levels with that of DEmiRs (Table 1). The ethylene biosynthesis-related *OsACO4* (Os11g0186900) gene was targeted by osa-miR531b (Table 1, Fig. 6A). *OsACO4* is one of 1-aminocyclopropane-1-carboxylic acid (ACC) oxidase (ACO) genes required for ethylene biosynthesis [39]. Interestingly, the expression of *OsACO4* was barely detected from embryo in 30 DAH and from all three endosperm samples; however, the expression level was gradually increased during 45 to 60 DAH in the embryo (Fig. 6B). Meanwhile, the expression level of *OsACO4*-targeting osa-miR531b was maintained from 30 to 45 DAH in the embryo and was down-regulated at 60 DAH. This change may allow the induction of *OsACO4* in the embryo and promote ethylene biosynthesis, which finally affects the increased PHS rate at 60 DAH by reducing the ABA sensitivity. Based on previous knowledge that the transcript level of ACO is inhibited by ABA [13], it is tempting that osa-miR531b supports the role of ABA by inhibiting *OsACO4* at the post-transcriptional level in the embryo and endosperm during seed maturation (between 30 and 45 DAH).

**Table 1:** List of DEmiRs and their predicted target genes exhibiting anti-correlated differential expression changes with DEmiRs

miRNA ID	Log_2_FC (miRNA, 60DAH/30DAH)	Target ID (RAP-DB)	Pairing Score	Description/Gene Name	Log_2_FC (Gene, 60DAH/30DAH)
osa-miR408-3p	-1.02	Os01g0860450	4.0	Hypothetical protein.	1.45
osa-miR528-5p	-1.56	Os01g0762000	3.5	*OspPLAIValpha*	1.22
		Os01g0519050	4.0	Hypothetical gene.	1.76
osa-miR531b	-3.17	Os01g0589000	3.0	Nucleic acid-binding, OB-fold domain containing protein.	1.19
		Os06g0308000	3.0	*OsTIG*	1.23
		Os11g0186900	3.0	*OsACO4*	4.43
		Os05g0521500	3.5	Peptidase S16, lon N-terminal domain containing protein.	1.15
		Os07g0538000	3.5	1-3,1-4-beta-glucanase.	3.56
		Os11g0507200	4.0	Similar to transferase.	3.24
osa-miR1846a-5p	-1.46	Os03g0561400	3.5	Conserved hypothetical protein.	2.02
		Os03g0203200	4.0	HTD2|D88|D14	1.61
osa-miR1848	-4.18	Os08g0412700	2.5	Protein of unknown function DUF1262 family protein.	1.48
		Os01g0321300	3.0	Similar to Protein translocase subunit secA.	1.06
		Os03g0267100	3.0	Hypothetical protein.	1.32
		Os06g0114366	3.0	Pentatricopeptide repeat domain containing protein.	1.07
		Os05g0522600	3.5	Leucine-rich repeat, plant specific containing protein.	1.42
		Os07g0538000	3.5	1-3,1-4-beta-glucanase.	3.56
		Os07g0572100	3.5	*DAO*	2.76
osa-miR5493	-2.72	Os01g0926400	4.0	Similar to Pectin-glucuronyltransferase.	1.11
		Os03g0693900	4.0	*OsOXO3|OsGLP3-5*	7.58
		Os03g0694000	4.0	*OsOXO4|OsGLP3-6*	6.56
osa-miR5504	-2.06	Os12g0169950	4.0	Hypothetical gene.	1.36
osa-miR820c	-0.72	Os07g0687900	4.0	*OsGolS2|wsi76*	1.73
osa-miR166i-5p	1.16	Os01g0607900	2.0	*OsRPK1*	-3.35
		Os04g0435500	3.0	*OsTCHQD1*	-1.97
		Os01g0263300	3.5	*OsPOX1|ddOs319*	-1.75
		Os08g0127500	3.5	Acid phosphatase/vanadium-dependent haloperoxidase related family protein.	-1.31
		Os06g0705000	4.0	*OsGMT1/OsGT64A*	-1.45
osa-miR171h	2.96	Os03g0302800	1.5	Similar to Long cell-linked locus protein.	-8.69
		Os03g0184500	4.0	Transcriptional factor B3 family protein.	-1.25
osa-miR319a-3p	2.13	Os02g0628600	3.0	*ARF8*	-2.64
		Os08g0442700	3.0	*COE1*	-3.31
		Os03g0204300	4.0	Conserved hypothetical protein.	-1.34
		Os03g0591300	4.0	Helix-loop-helix DNA-binding domain containing protein.	-4.16
		Os08g0561500	4.0	Similar to nodulin-like protein 5NG4.	-2.37
		Os11g0539000	4.0	*OsSTA262*	-1.09
osa-miR390-5p	0.92	Os03g0314750	3.0	Hypothetical conserved gene.	-3.00
		Os08g0410266	3.5	Protein of unknown function DUF295 domain containing protein.	-1.52
		Os08g0446400	3.5	Leucine-rich repeat, N-terminal domain containing protein.	-1.33
		Os04g0571600	4.0	*OsMATE19*	-6.41
osa-miR437	1.16	Os01g0872000	2.5	*NPF5.21*	-3.45
		Os02g0762100	2.5	4-hydroxy-4-methyl-2-oxoglutarate aldolase	-1.09
		Os06g0657500	3.0	*PLT2*	-1.17
		Os05g0566400	3.5	*OsMPK7|OsMAPK20-5*	-2.25
		Os02g0186200	4.0	Similar to Cytochrome P450 CYP71K14.	-1.05
osa-miR1861h	3.10	Os01g0733500	4.0	*RD22| OsBURP3*	-1.30
osa-miR2090	1.91	Os03g0302200	3.0	*OsHIRP1*	-2.32
		Os04g0453400	4.0	*OsSTP17*	-1.68
		Os05g0486300	4.0	Similar to CCR4-NOT transcription complex subunit 2.	-1.58
osa-miR5827	1.50	Os07g0568700	3.0	*OsFOR1|PGIP*	-1.96
		Os03g0728900	3.5	Helix-loop-helix DNA-binding domain containing protein.	-1.13
		Os01g0661700	4.0	Hypothetical gene.	-3.54
		Os02g0579800	4.0	*OsFWL2*	-1.22
		Os02g0756600	4.0	Phosphate-induced protein 1	-2.36
		Os07g0252400	4.0	*OsCESA6*	-3.02
		Os08g0144100	4.0	*CML32*	-4.25

The table represents log_2_ fold change (log_2_FC) values of DEmiRs and their predicted target genes during the 30 to 60 DAH transition in the embryo

*OsMATE19* (Os04g0571600), a multi-antimicrobial extrusion protein, was predicted to be targeted by osa-miR390-5p (Table 1, Fig. 6C). *OsMATE19* is one of the four predicted rice homologs of *AtDTX50*, which functions as an ABA exporter in *Arabidopsis* [40]. The seeds of *dtx50* exhibit slow germination and a low ABA-sensitive germination rate; therefore, an increase in osa-miR390-5p in the embryo may contribute to the down-regulation of the *OsMATE19* transcript level and affect the change in the dormancy level during seed maturation (Fig. 6D). Notably, *OsMATE19* was also predicted to be targeted by osa-miR166b-3p (Additional file 1: Fig. S3). Though the expression level of osa-miR166b-3p in the embryo did not change significantly (p.adj > 0.05), it was the most abundantly expressed miRNA among those observed in our dataset; moreover, a gradual increase in the level was observed in the embryo (Additional file 1: Fig. S3). Additionally, after searching the degradome dataset from TarDB [41], we found experimental evidence that the predicted target site of osa-miR166b-3p on *OsMATE19* is targeted by the osa-miR166 family, which has the same or extremely similar sequence as osa-miR166b-3p. However, osa-miR390-5p was not detected from the *OsMATE19*-targeting miRNA list in TarDB, possibly due to its relatively low expression or the lack of a proper degradome dataset. Thus, the results of the analysis indicated the biological roles of several miRNAs in targeting ethylene and the ABA signaling-related genes, which can affect the changes in the seed dormancy level in the rice embryo during the 30 to 60 DAH maturation stage.

## Discussion

Seed maturation is directly related to dormancy for the establishment of the next generation [2, 3]. Therefore, transcriptome and miRNAome analyses were conducted in this study to explore the dynamics of seed dormancy during maturation in rice (*Oryza sativa* L. cv. Nipponbare) seeds. Based on our previous study [27], we conducted the experiment by dividing the seeds into embryos and endosperms and categorizing them into three maturation stages (30, 45, and 60 DAH). The PHS rates of Nipponbare seeds significantly increased at 60 DAH compared to those at 30 and 45 DAH (Fig. 1A, B). This phenomenon may have been caused by specific genes and miRNAs from 45 to 60 DAH or 30 to 60 DAH, which may have affected the seed dormancy.

In our results, the 500-grain weight peaked at 30 DAH. In addition, no significant changes were observed in the 500-grain weight during the following stages, indicating that seed development and filling concludes at 30 DAH and transitioning into the subsequent seed maturation stages (Additional file 1: Fig. S4A). Interestingly, the ABA content in the embryos decreased gradually during maturation, and the IAA content decreased after 30 DAH (Additional file 1: Fig. S4B, C). In addition, in our transcriptome data, the expression levels of *OsSdr4*, *OsVP1*, and *OsDOG1L-3* which are known to regulate the seed dormancy in relation to ABA [42–44], corresponded closely to the three maturation stages in the embryo. The expression levels of *OsSdr4*, *OsVP1*, and *OsDOG1L-3*, which positively control seed dormancy via ABA signaling, decreased at 60 DAH compared to those at 30 and 45 DAH. However, no significant changes in the expression of the aforementioned genes were observed based on the maturation stages in the endosperm, and the transcript per million mapped (TPM) values were notably lower compared to those in the embryo (Additional file 1: Fig. S4D). These results were consistent with the PHS ratio (Fig. 1B), indicating that our omics data from each maturation stage in the seed dormancy-related omics analyses were appropriate. In addition, our results showed the decrease in IAA content after 30 DAH in the embryo (Additional file 1: Fig. S4C). In previous studies, it was discovered that the auxin level is involved in the interaction between *ARF10/16*, *ABI3* [9], and auxin-responsive gene *SAUR32* is involved in ABA accumulation [11], regulating dormancy in *Arabidopsis*. However, in rice, little is known about interaction of auxin and ABA contents, auxin-, and ABA-responsive genes in seed dormancy. To illuminate this, our study supports the notion that genetic changes affecting seed dormancy primarily occur during maturation in the embryo, along with the gradual decrease in ABA content during seed maturation and the decrease in IAA content after 30 DAH. In the endosperm, the ABA content was significantly lower compared to the embryo, and no significant changes were observed during seed maturation (Additional file 1: Fig. S4B). This result further highlights the primary role of the embryo in seed dormancy. Additionally, the IAA content is maintained at a higher level in the endosperm compared to the embryo, suggesting the involvement of the endosperm in preserving the seed shape and quality, as discussed in a previous study [45].

Interestingly, the DEGs of the embryo between 30 and 45 DAH showed GO terms related to meiosis, mitosis, and photosynthesis. According to a previous study [46], a proper cell cycle related to meiosis and mitosis is critical for cell growth and cell expansion in the seed. Therefore, cell growth and expansion may be active in the embryo during the early maturation stage, unlike during zygotic embryogenesis. Furthermore, in the case of green embryos found in *Arabidopsis* and beans during early development, embryonic photosynthesis might provide oxygen to the seed and increase the energy supply, thereby facilitating development [47, 48]. However, this process is not well understood in rice seeds. The GO analysis in the current study revealed photosynthesis-related terms in the embryo at the early maturation stage (Fig. 2D). These findings indicate that green embryos in rice can engage in photosynthesis to support seed growth at the early maturation stage, similar to *Arabidopsis* and beans. In summary, we propose that green embryos in the early maturation stage, following zygotic embryogenesis, can engage in photosynthesis to provide energy for the growth of the seed cell. During the subsequent seed maturation, hormone-related genes in the embryo, rather than those in the endosperm, play a role in dormancy. In addition, auxin- and ethylene-related *OsSAUR6*, *OsSAUR12*, *OsSAUR23*, *OsSAUR25*, and *OsERF087* were identified as new genes implicated in seed dormancy during maturation. Especially, considering the lower accumulation of ABA content in the *saur32* mutant compared to WT *Arabidopsis* in the previous study [11], the gradual decrease of ABA content in the embryo during maturation closely corresponds to the gradual decrease in the expression level of *OsSAURs* (Fig. 4D, Additional file 1: Fig. S4B). In addition, the gradual decrease in the expression level of *OsSAURs*, the auxin-responsive genes, might also be associated with a decrease in IAA content after 30 DAH, potentially playing an important role in determining dormancy during maturation (Fig. 4D, Additional file 1: Fig. S4C). Further research on auxin-responsive *OsSAURs* and their interaction with auxin and ABA in the embryo during maturation may illuminate novel models concerning auxin, ABA, and seed dormancy in rice.

Through profiling and target prediction of DEmiRs during seed maturation, we suggested a novel, putative regulatory relationship between osa-miR531b and *OsACO4*, as proposed in a previous study [49]. Osa-miR531b was reported as a member of the drought-responsive miRNA family exhibiting down-regulated expression levels in rice treated with drought stress [50]. It is suspected that the down-regulation of osa-miR531b in the embryo is caused by desiccation because seeds are known to undergo desiccation during maturation. *OsACO1*, one of the annotated genes in the rice ACO family, was down-regulated during the 45 to 60 DAH transition in the embryo; this is opposite to that seen with *OsACO4* during the same period (Additional file 3: Dataset S1). Given that ethylene is required for seed germination, the above-mentioned interchange in the expression level between *OsACO1* and *OsACO4* during seed maturation in the embryo indicates the putative, stage-specific contribution of *OsACO4* in increasing the PHS at 60 DAH, possibly by producing ethylene in embryo. Follow-up studies are required to validate the organ- and stage-specific functions of *OsACO4* in rice.

## Conclusions

In this study, spatio-temporal profiling of the transcriptome and small RNAome were conducted in the embryo and endosperm of *Oryza sativa* cv. Nipponbare seeds during the three maturation stages. The global transcriptome dynamics observed in the embryo were consistent with the changes in the PHS rate but differed from the dynamics observed in the endosperm transcriptome. In-depth investigations of the embryo transcriptome revealed that the dynamics in the embryo during the 45 to 60 DAH and 30 to 60 DAH transitions are mainly associated with changes in various phytohormone signaling pathways, including GA, ABA, auxin, and ethylene (Figs. 2, 3 and 4, Additional file 2: Tables S7, S9, and S10). This finding suggested the presence of complex cross-talks between the phytohormones in the embryo, which may influence seed germination and dormancy during the 45 to 60 DAH and 30 to 60 DAH transitions. Additionally, four newly discovered auxin-related *SAUR* genes and one ethylene-related *ERF* gene, which are expected to play a role in regulating seed dormancy, were profiled in this study. The profiling of the miRNAome and the prediction of DEmiRs in the embryo indicated two potential miRNA-target modules, osa-miR531b – *OsACO4* and osa-miR390-5p – *OsMATE19*, which may be associated with the regulation of ethylene biosynthesis and ABA transport in rice seeds. The findings of this study will expand our knowledge about the transcriptome/small RNAome dynamics and their effects on the regulation of seed dormancy during maturation.

## Methods

### Plant materials, growth conditions, grain weight measurement and PHS assay

Wild-type Nipponbare (*Oryza sativa* L. cv. Nipponbare) was bred in an experimental field at the Seoul National University, Suwon, Republic of Korea. In our previous study [27], we defined three seed maturation stages (30, 45, and 60 DAH) based on PHS rates in the rice seeds of PHS-susceptible accession and PHS-resistant accessions, which exhibited different seed dormancy characteristics. Therefore, Nipponbare seeds and panicles were harvested at 30, 45, and 60 DAH and sampled by dividing them into the embryo and endosperm (Fig. 1C). The whole seeds, excluding seed coat, were freshly harvested and frozen in liquid nitrogen at each maturation stages. The 500-grain weight were measured and replicated 3 times. Nine freshly harvested panicles were incubated for 7 days at 25℃ and 100% relative humidity for the PHS assay. The number of germinated seeds in each panicle was recorded and expressed as a percentage of the total number of seeds per panicle, as described previously [27]. Nine replicates were used at each time point. Statistical analysis was performed using the Student’s *t*-test (^***^P < 0.001).

### Quantification of endogenous ABA and IAA content

The ABA and IAA in the embryos and endosperms of Nipponbare at each maturation stages were extracted following the method in Salem et al. [51]. Each frozen sample, when homogenized into a powder, weighed approximately 50 mg for hormone extraction. The endogenous levels of ABA and IAA were determined by NICEM (Seoul, Republic of Korea) using the TSQ Altis Rapid Liquid Chromatography Tandem Mass (LC/MS/MS) Spectrometer (Thermo Fisher Scientific, United States). Three replicates were used at each time point. Statistical analysis was performed using the Student’s *t*-test.

### Construction of mRNA-Seq and small RNA-Seq libraries

The embryos and endosperms at each stage were separately sampled from 10 seeds obtained from 12 individual plants (*n* = 120 seeds), at three maturation stages (30, 45, and 60 DAH), for one biological replicate. Subsequently, 36 plants (12 plants per replicate) at each maturation stage were used for three replicates. TRIzol Reagent (Invitrogen, United States) was used for RNA extraction, as described previously [27]. For mRNA-Seq library construction, total RNAs extracted from three biological replicates of embryos and endosperms were used. The mRNA-Seq libraries were constructed from 2 μg of total RNA using the SENSE mRNA-Seq Library Pep Kit V2 for Illumina platforms (LEXOGEN, Austria), according to the manufacturer’s instructions. For small RNA-Seq library construction, total RNAs obtained from three biological replicates of embryos and two biological replicates of endosperms were employed. Small RNAs between 15- and 30-nt were isolated from 20 μg of total RNA by the size-fractionation using 15% Urea-PAGE gel, and the small RNA-Seq libraries were constructed using the Small RNA-Seq Library Prep Kit for Illumina Platforms (LEXOGEN, Austria), according to the manufacturer’s instructions.

### mRNA-Seq data processing and differential expression analysis of genes

In total, nine mRNA-Seq datasets for embryo (three biological replicates for each stage) and nine mRNA-Seq datasets for endosperm (three biological replicates for each stage) were analyzed in this study. mRNA-Seq was performed using the Illumina HiSeq 2500 platform. Raw reads were processed using FastQC, and low-quality reads and adapters were removed using Trimmomatic (v0.3.6) [52]. The remaining reads were aligned to the rice genome (IRGSP-1.0) using Hisat2 with default parameters [53]. FeatureCounts was used to calculate the read counts mapped to each gene expression level [54]. DESeq2 was used to identify DEGs (*p* value < 0.05; log_2_FC > ± 1) [55]. Significantly enriched GO [56] and KEGG pathway [57] terms were selected with the fold enrichment and FDR using the GO Resource web server (http://geneontology.org) [56] and ShinyGO web server (http://bioinformatics.sdstate.edu) [58]. The MapMan software v3.6.0 was used to map the transcriptome data and identify significantly overrepresented functional genes [37]. A dataset containing the IDs of DEGs was constructed with “Regulation overview”.

### Quantitative real-time PCR

The cDNA was synthesized from each RNA sample using PrimeScript Reverse Transcriptase (Takara, Japan), according to the manufacturer’s instructions. Quantitative real-time PCR (qRT-PCR) was performed using the AccuPower 2X GreenStar qPCR Master Mix (Bioneer, Republic of Korea) with SYBR Green detection and gene-specific primers. The Ct values for the genes were obtained using Os03g0718100 (*OsACT1*) as a control, and the relative expression levels of target genes were determined using the ΔΔCt method [59]. The primer sequences of the genes in this study are listed in Additional file 2: Table S14. Statistical analyses were performed using Student’s t-test (**: *P* < 0.01; ***: *P* < 0.001).

### Small RNA-Seq dataset processing, differential expression analysis, and target gene prediction of miRNAs

In total, nine small RNA-Seq datasets for embryo (three biological replicates for each stage) and six small RNA-Seq datasets for endosperm (two biological replicates for each stage) were analyzed in this study. Adapter trimming, quality control, and 18-to-26-nt-length read selection were performed using Trimmomatic (v0.3.9) and Cutadapt (v4.1) [52, 60]. The processed reads were mapped to rRNA/tRNA/snRNA sequences (RNACentral, v17) using Bowtie (v1.3.0, -v 1 -m 0 -a) to remove the structural non-coding RNA reads [61, 62]. The remaining reads were aligned to the IRGSP-1.0 reference genome sequence using Bowtie (v1.3.0, -v 0 -m 0 -a); the aligned reads were re-aligned to the same reference sequence with ShortStack (v3.8.5, --align_only --mismatches 0 --mmap u --bowtie_m 1000 --ranmax 3) [63]. The miRNA read count was calculated by adding 20 ~ 24-nt aligned reads whose 5’ end position fell into -2 ~ + 1-nt from the annotated genomic location of the miRBase-enlisted known miRNAs using FeatureCounts [54]. Measured miRNA read counts were then subjected to the differential expression analysis using DESeq2 packages, and miRNAs satisfying a “p.adj < 0.05” were selected as DEmiRs and used for the downstream analysis. Hierarchical clustering and heatmap visualization of DEmiRs were performed after the log2-transformation of counts per million (CPM)-normalized miRNA expression values. The sequences of these miRNAs were subjected to psRNATarget (https://www.zhaolab.org/psRNATarget/) with a default option to predict the target genes of the differentially expressed miRNAs [38].

### Supplementary Information

**Additional file 1.** Figure S1-S4.

**Additional file 2.** Table S1-S14.

**Additional file 3.** Dataset S1-S2.

## Data Availability

The datasets supporting the conclusions of this article are available in the NCBI BioProject (PRJNA1020757). The statistical results supporting the conclusions of this article are included in Additional file 3.

## Abbreviations

DAH: Days after heading
PHS: Pre-harvest sprouting
DEG: Differentially expressed gene
DEmiR: Differentially expressed miRNA
GO: Gene Ontology
KEGG: Kyoto Encyclopedia of Genes and Genomes
ABA: Abscisic acid
GA: Gibberellic acid
IAA: Indole-3-acetic acid
WT: Wild-type
miRNA: MicroRNA
PCA: Principal component analysis
Log_2_FC: Log_2_ fold change
qRT-PCR: Quantitative real-time polymerase chain reaction
