# Supplementary material for: Analysis of the global transcriptome and miRNAome associated with seed dormancy during seed maturation in rice (Oryza sativa L. cv. Nipponbare)
Source: BMC Plant Biol. 2024 Mar 26;24:215. doi: 10.1186/s12870-024-04928-6 (PMC10964676; doi:10.1186/s12870-024-04928-6)
Supplement: Supplementary file 1 — Additional file 1. Figure S1-S4. [file 12870_2024_4928_MOESM1_ESM.pdf]

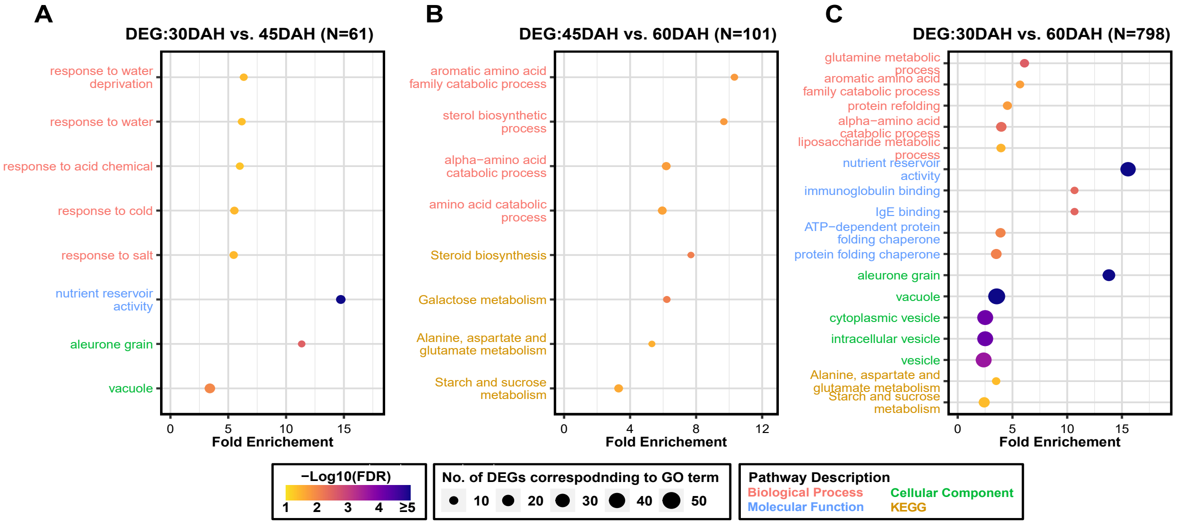

Figure S1. Gene Ontology (GO) and Kyoto Encyclopedia of Genes and Genomes (KEGG) terms in differentially expressed genes (DEGs) in the endosperm. **(A-C)** GO and KEGG enrichment terms based on DEGs between 30 and 45 DAH **(A)**, 45 and 60 DAH **(B)**, and 30 and 60 DAH **(C)**. The Y-axis represents the enriched GO and KEGG terms. The X-axis represents the amount of fold enrichment of GO and KEGG pathway terms. The top five GO terms associated with "biological process", "molecular function", and "cellular component", and the top five KEGG pathway terms based on fold enrichment > 2 and false discovery rate (FDR) < 0.05 were selected. 30 DAH vs. 45 DAH: DEGs between 30 and 45 DAH. 45 DAH vs. 60 DAH: DEGs between 45 and 60 DAH. 30 DAH vs. 60 DAH: DEGs between 30 and 60 DAH. The enriched KEGG pathway terms were obtained using ShinyGO.

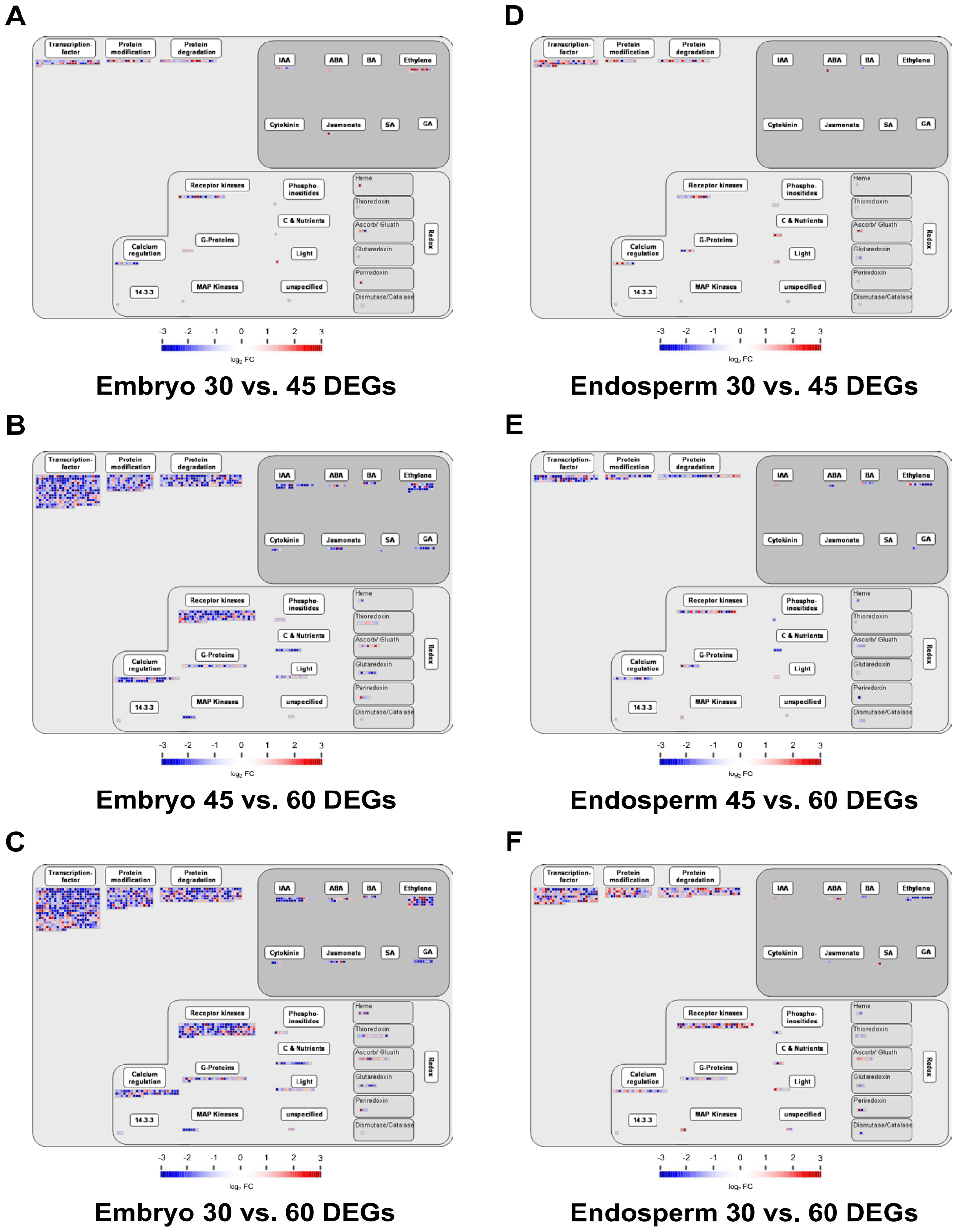

Figure S2. "Regulation overview" in DEGs of the embryo and endosperm using MapMan. **(A-F)** "Regulation overview" of the embryo 30 vs. 45 DEGs **(A)**, 45 vs. 60 DEGs **(B)**, 30 vs. 60 DEGs **(C)**, endosperm 30 vs. 45 DEGs **(D)**, 45 vs. 60 DEGs **(E)**, and 30 vs. 60 DEGs **(F)**.

A

```
5'-GGUGAACGAAGCCUGGUCCGU-3' Os04g0571600:400-420nt
   :: ::: :::::::::::::      (OsMATE19)
3'-CCCCUUACUUCGGACCAGGCU-5' osa-miR166b-3p
   CUCCUUACUUCGGACCAGGCU-5' osa-miR166h-3p
```

B

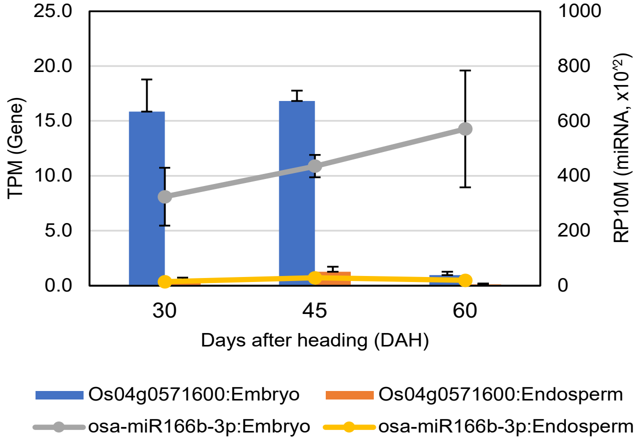

Figure S3. osa-miR166 targets *OsMATE19*. **(A)** Schematic representation for the pairing between osa-miR166 and *OsMATE19* transcript. **(B)** Profiled expression levels of osa-miR166 and its predicted target gene, *OsMATE19*. TPM: transcript per million mapped, RP10M : reads per ten million mapped.

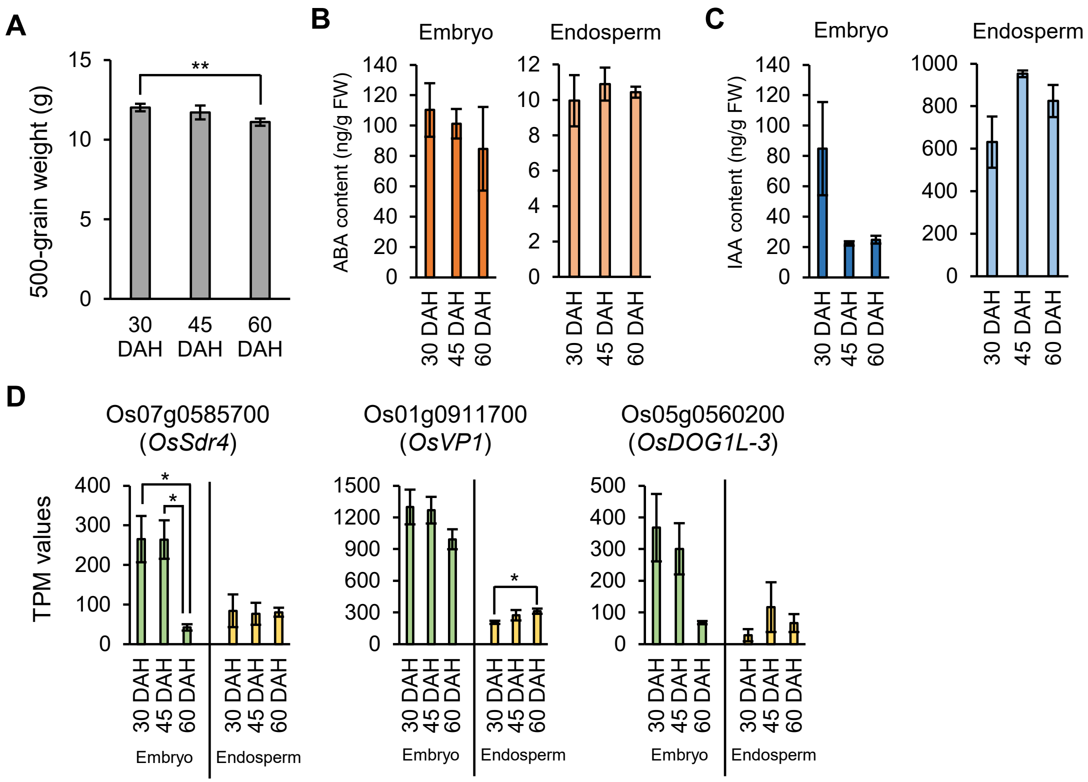

Figure S4. 500-grain weight, ABA, IAA contents, and transcript per million (TPM) values of *OsSdr4*, *OsVP1*, and *OsDOG1L-3* at each maturation stage. **(A)** 500-grain weight at 30, 45, and 60 DAH. **(B-C)** ABA content **(B)** and IAA content **(C)** in fresh weight (FW) of the embryo and endosperm at 30, 45, and 60 DAH. **(D)** TPM values of *OsSdr4*, *OsVP1*, and *OsDOG1L-3* in the embryo and endosperm at 30, 45, and 60 DAH. Data represent the mean ± standard error of mean (SEM; $N = 3$). The significance was determined using Student's *t*-test, $*P < 0.05$ and $***P < 0.001$.
